# The Impact of Protease during Recovery from Viable but Non-Culturable (VBNC) State in *Vibrio cholerae*

**DOI:** 10.3390/microorganisms9122618

**Published:** 2021-12-18

**Authors:** Anusuya Debnath, Shin-ichi Miyoshi

**Affiliations:** Graduate School of Medicine, Dentistry and Pharmaceutical Sciences, Okayama University, 1-1-1, Tsushimanaka, Kita-ku, Okayama 700-8530, Japan; miyos-s@okayama-u.ac.jp

**Keywords:** VBNC, recovery, proteinase K, growth, protease

## Abstract

*Vibrio cholerae* can survive cold stress by entering into a viable but non-culturable (VBNC) state, and resuscitation can be induced either by temperature upshift only or the addition of an anti-dormancy stimulant such as resuscitation-promoting factors (Rpfs) at suitable temperature. In this study, the role of proteinase K was analyzed as an Rpf in *V. cholerae*. A VBNC state was induced in *V. cholerae* AN59 in artificial seawater (ASW) media at 4 °C, and recovery could be achieved in filtered VBNC microcosm, called spent ASW media, merely by a temperature upshift to 37 °C. The resuscitation ability of spent ASW was further enhanced by the addition of proteinase K. The mode of action of proteinase K was investigated by comparing its effect on the growth of the VBNC and culturable state of *V. cholerae* in ASW and spent ASW media. The presence of proteinase K allowed culturable cells to grow faster in ASW by reducing the generation time. However, this effect of proteinase K was more pronounced in stressed VBNC cells. Moreover, proteinase K-supplemented spent ASW could also accelerate the transition of VBNC into recovered cells followed by rapid growth. Additionally, we found that dead bacterial cells were the substrate on which proteinase K acts to support high growth in spent ASW. So, the conclusion is that the proteinase K could efficiently promote the recovery and growth of dormant VBNC cells at higher temperatures by decreasing the duration of the initial lag phase required for transitioning from the VBNC to recovery state and increasing the growth rate of these recovered cells.

## 1. Introduction

The viable but non-culturable state is a distinctive survival strategy adopted by numerous microbial species to habituate in unfavorable environmental conditions. The inability to detect various pathogenic bacteria in the VBNC state makes them a serious threat for food safety and public health because there is always a possibility of their resuscitation under appropriate conditions. Vibrio species are the predominant inhabitants of the marine ecosystem, and several of them were reported to be able to convert into the VBNC state when kept under low temperature and nutrient starvation conditions [1]. Interestingly, some species of *Vibrio* such as *Vibrio splendidus* prefer lower winter temperatures than other species [2]. It has been reported that the mere transfer from low temperature to high temperature could induce recovery in *Vibrio cholerae*, *V. parahaemolyticus* and *V. vulnificus* [3,4,5,6]. However, recovery from the VBNC state has been addressed different hypotheses; for example, according to Nilsson et al. and Whitesides et al., all the population of *V. vulnificus* in a microcosm become VBNC and resuscitate without cell division. In the case of *V. cholerae*, Ravel et al. supported the concept that the recovered population resulted from the growth of culturable cells, but some studies suggest that few VBNC cells retain their ability to grow and imitate the resuscitation of an entire population [3,7].

Apart from high temperature-induced resuscitation, several other methods were discovered for recovery from the VBNC state. The pathogenic strain of *Escherichia coli* O157:H7 can be recovered using the autoinducer AI-2 produced during biofilm formation in a serum-based media [8]. The resuscitation in *E. coli* can also be supported by a concoction of amino acids such as methionine, glutamine, threonine, serine and asparagine [9]. In *Salmonella enteritidis*, it has been shown that sodium pyruvate could restore the biosynthesis of DNA and protein in VBNC cells [10]. Catalase was shown to be a catalyst for resuscitation in *S. enterica* serovar Typhi [11], *S. enteritidis* [12] and *Vibrio cholerae* [13]. Few studies have shown that direct interaction with eukaryotic cells can also facilitate resuscitation. Senoh et al. found that low temperature-induced VBNC cells of different enteric pathogenic species could be converted to the culturable state by co-culture with several eukaryotic cell lines [14]. The inoculation of VBNC cells into an embryonated egg was shown to be an efficient method to recover from the dormant state in *Listeria monocytogenes* [15] and *Campylobacter jejuni* [16].

Another class of secretory molecule, a bacterial cytokine known as resuscitation-promoting factor (Rpf), was identified that stimulates resuscitation and growth in *Micrococcus luteus* and *Mycobacterium* spp. [17,18]. Rpfs of Gram-positive bacteria possess a muralytic activity that is predicted to be responsible for the hydrolysis of peptidoglycan and cell division [16,17]. The Rpfs of Gram-negative bacteria known as YaeZ belong to a different class of protein and are essential for both survival and exit from the VBNC condition, as reported for *S. enterica* serovar Oranienburg and *E. coli* [19,20]. YaeZ of *V. parahaemolyticus* is reported to be a classic actin-like nucleotide binding protein and essential for recoveryfrom the VBNC state [21].A recent report on Rpf of *Vibrio harveyi* revealed that the resuscitation-inducing ability of YaeZ was dependent on its proteolytic activity and independent of muralytic activity [22].

It was shown in *Staphylococcus aureus* that the protease treatment of spent culture supernatant could reduce its resuscitation effect [23], whereas a protease was shown to be an Rpf in *V. harveyi* [22]. In contrast, Pinto et al. suggested that the breakdown products of Rpf obtained by proteinase K treatment of spent culture supernatant could also restore VBNC cells of *E. coli* [9]. So, the role of proteinase K as a potential Rpf and its probable mode of action was analyzed in this study. The outcome of this study might be beneficial for the detection of VBNC cells.

## 2. Materials and Methods

### 2.1. Bacterial Strains and Growth Conditions

In this study, *V. cholerae* non-O1/non-O139 strain AN59 isolated from a brackish environment in Okayama was used [3]. Bacteria were grown in Luria–Bertani (LB) broth (1% tryptone, 0.5% yeast extract and 1% NaCl) at 37 °C for 18 h, and the culturable cell count was estimated by plating on nutrient agar (NA) plates (BD Difco, Franklin Lakes, NJ, USA).

### 2.2. Viable but Non Culturable (VBNC) State

To experimentally induce the VBNC state, the late-log phase bacterial culture was 100-fold diluted in 30 mL ASW media in multiple sets and incubated at 4 °C [24]. The culturable cell count was determined at an interval of 7days by plating 0.1 mL of the suspension on NA plates. A the time point at which 0.1 mL gave no culturable cell count on the NA plate, 10 mL of the VBNC sample was used instead of 0.1 mL. The 10 mL VBNC microcosm was filtered through 0.22 μm membrane filters (Millipore, Burlington, MA, USA), and the filter was placed on the NA plate for 32 h at 37 °C. When the culturable cell count became ≤1cfu mL^−1^, it was considered to represent the VBNC state [25].

### 2.3. Recovery State

After entry into the VBNC state, a 10 mL aliquot of VBNC cells from the 30 mL VBNC microcosm was shifted from 4 °C to 37 °C for recovery on days 1, 7, 14 and 21 to check the length of the recovery period. It was kept at 37 °C in a stationary position, and the recovered cell count (cfu mL^−1^) was determined. Simultaneously, another 10 mL aliquot was used to determine the culturable cell count of the VBNC microcosm at these time points.

The spent ASW media was obtained from the original VBNC microcosm by filtration through a 0.22 μm membrane filter. The presence and nature of resuscitation-promoting factor (Rpf) in spent ASW media was analyzed by subjecting it to heat and protease treatment. Nine milliliters of spent ASW media was used for heat treatment at 100 °C for 10 min and then inoculated with 1ml of VBNC cells; i.e., 10-fold diluted VBNC cells. For the induction of recovery, this 10 mL culture was incubated at 37 °C for 32 h. In another set, 100 μg ml^−1^ proteinase K was added into 9 mL of spent ASW media, inoculated with 1 mL of VBNC cells and incubated at 37 °C for 32 h. After incubation, the recovered cell count was determined using the plate count method. In some recovery experiments, trypsin and subtilisin A were used. The term “protease” was used for serine protease in general.

### 2.4. Culturable State

The late-log phase bacterial culture was 100-fold diluted in 30 mL ASW media and incubated at 37 °C for 16 h to acclimatize in ASW. On the next day, it was 10^5^-fold diluted, and 1mL of culturable cells was added to 9 mL of suitable media so that the final cell concentration would become 10 cfu mL^−1^.

### 2.5. Dead Cell Preparation

In total, 100 µL (10^7^ cfu) of late-log phase culture was centrifuged and washed, and the pellet was dissolved in 100 µL of 2.5 M NaCl and incubated at 37 °C for 2 h, which led to the complete killing of *V. cholerae,* as checked by the plating method. Then, 100 µL of dead cell was added into ASW media, which increased the NaCl molarity from 423 mM to 448 mM in the final volume of 10 mL. This change in molarity did not affect the growth or viability of *V. cholerae* in ASW.

### 2.6. Growth in ASW and Spent ASW Media

The growth of culturable cells and VBNC cells was checked in 10 mL of different types of media such as ASW, spent ASW, ASW supplemented with proteinase K and spent ASW supplemented with proteinase K. An initial inoculum of 10 cfu mL^−1^ was used for culturable cells and 0.08 to <0.01 cfu mL^−1^ for VBNC cells. After inoculation, the culture was kept at 37 °C for up to 32 h in a stationary position, and the cell count was checked at 0 h and 32 h time points or at different time points by plating the appropriate dilution on NA plates.

### 2.7. RNA Isolation and Real Time RT-PCR

For quantitative real-time reverse transcription polymerase chain reaction (qRT-PCR) experiments, the RNA was isolated from culturable and recovery states of *V. cholerae* cells. Since, RNA was purified at 2 h, 4 h and 8 h after recovery induction, there was no visible pellet. Therefore, the sample was filtered through a 0.22 μm membrane filter to collect the cells, and RNA was extracted as mentioned previously [3]. For RT-PCR, we used aLuna^®^ Universal One-Step kit RT-qPCR kit (NEB, Ipswich, MA, USA) with 50 ng of RNA in a Mini Opticon Real-time PCR system (BIO-RAD, Hercules, CA, USA). The relative mRNA expression of the protease genes such as *vc0099*, *vc1200, vca0803* and *vc1989 (yaeZ)* was calculated using *dnaK* as an internal control at time points of 2 h, 4 h and 8 h after the temperature upshift. The list of primers is provided in Table S1.

### 2.8. Skim Milk Plate Assay

Two types of skim milk (Wako Chemicals, Richmond, VA, USA) plate were prepared using ASW and spent ASW media. We added 0.2 g of skim milk to 10 mL of dH_2_O, and 0.6 g of agar was added to 30 mL ASW, followed by heating at 100 °C for 10 min. These two solutions were then mixed to make a 1% skim milk ASW plate. Another type of plate was prepared by mixing two sterile solutions consisting of 0.4 g of skim milk in 10 mL of dH_2_O and 0.6 g agar in 10 mL of ASW media. This 20 mL mixture was cooled down to 50 °C, and then 20 mL of spent ASW was added, as high temperature could destroy the resuscitation effect of spent ASW. The 40 mL solution was poured into a Petridish to give 1% skim milk spent ASW plate. A well was created of 8 mm in diameter, and 200 µL of VBNC sample was loaded followed by incubation at 37 °C for 96 h.

### 2.9. Statistical Analysis

The data were analyzed by Student’s *t*-test. A probability level (*p*) value of ≤ 0.05 was taken as statistically significant. The data were represented as means ± SE of three independent events.

## 3. Results

### 3.1. The Effect on Resuscitation Ability of V. cholerae VBNC Cells Either in Heat-Treated or Protease-Supplemented Spent ASW

**Figure 1:**
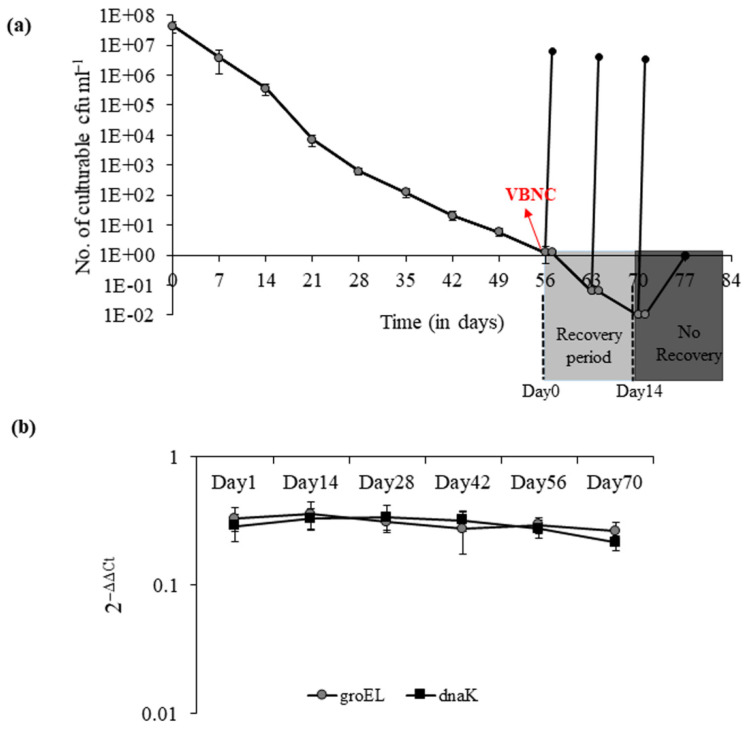
Progress curve of *V. cholerae* culturability and viability during the course of VBNC induction.(**a**) Culturable cell count was determined for *V. cholerae* AN59 during the induction of VBNC state at 4 °C (●) in ASW. Recovery was induced on days 56, 63, 70 and 77 after entry into VBNC state by temperature upshift from 4 °C to 37 °C (●). Each bar represents the mean ± SE of three independent experiments. (**b**) The relative mRNA expression of transcripts of molecular chaperones (**■**) *dnaK* and (●) *GroEL* were analyzed during the course of VBNC induction as an indicator of cell viability.

The *V. cholerae* strain AN59 took 56 days to enter the VBNC state when incubated at 4 °C under nutrient starvation conditions in ASW. It showed a gradual decrease in culturability from 4.2 × 10^7^ cfu mL^−1^ to ≤1 cfu mL^−1^ (Figure 1a). The stable mRNA expression of *groEL* and *dnaK* throughout the VBNC induction period was used as an indicator of viability for VBNC cells (Figure 1b). After entry into the VBNC state, the recovery was observed for a period of two weeks (between days 56 to 70) by a temperature upshift from 4 °C to 37 °C. After overnight recovery at elevated temperature, the recovered cell count was approximately 6 × 10^6^ cfu mL^−1^.

In order to understand the nature of Rpf, spent ASW was subjected either to heat (100 °C for 10 min) or protease (100 μg mL^−1^ of proteinase K) treatment. The heat treatment of spent ASW media inhibited the recovery of VBNC cells, whereas the addition of proteinase K did not. This shows that the Rpfs were heat-sensitive but protease-resistant (Table 1). Interestingly, the addition of proteinase K at the time of the temperature transition from 4 °C to 37 °C led to an extension in the total recovery period from a period of 2 weeks to 5 weeks—i.e., from day70 to day91—but not beyond that.

### 3.2. Effect of Proteinase K during Recovery of V. cholerae from VBNC State

**Figure 2:**
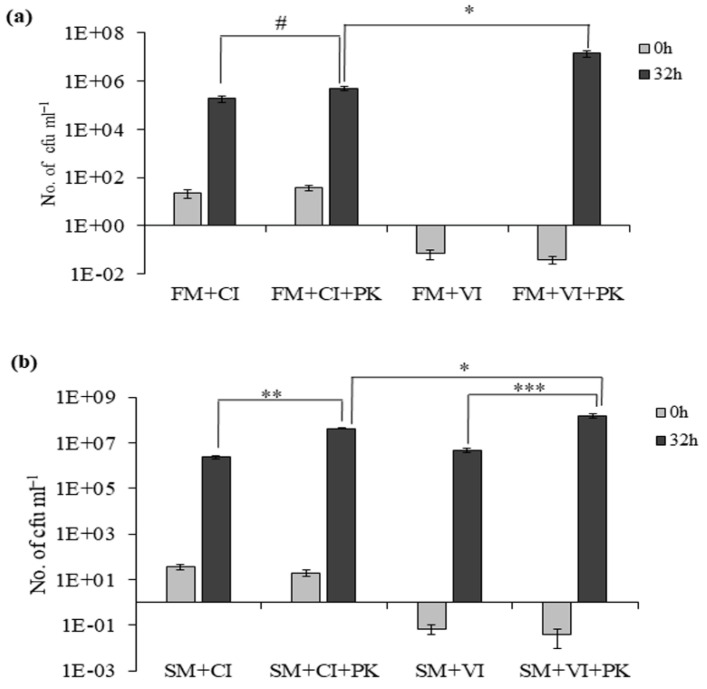
Effect of proteinase K on VBNC and culturable cells in ASW and spent ASW media. (**a**) Growth of culturable cells (CI) and recovery of VBNC cells (VI) in ASW media (FM) without or with 100 μg mL^−1^ of proteinase K (PK); cell count was determined at 0 h and 32 h. # represents non-significant difference in growth of CI between FM and PK-supplemented FM. Asterisk (*) represents statistically significant difference between the growth of CI and VI in FM + PK (*p* < 0.05). (**b**) Growth of CI cells and recovery of VI cells in SM (spent ASW media) and SM+PK. Each bar represents the mean ± SE of three independent experiments. Asterisk (**) represents statistically significant difference in growth of CI between SM and SM+PK. (*p* < 0.001).Asterisk (***) represents statistically significant difference in growth of VI between SM and SM+PK. (*p* < 0.02). Asterisk (*) represents statistically significant difference in growth between CI and VI in SM+PK (*p* < 0.05).

The VBNC cells in spent ASW supplemented with proteinase K led to an increase to 1.58 × 10^8^ cfu mL^−1^ after 32 h of incubation at 37 °C, whereas resuscitation without proteinase K led to a recovered cell count of 4.9 × 10^6^ cfu mL^−1^. The growth of culturable cells and VBNC cells in ASW media (FM) and proteinase K-supplemented ASW media (FM + PK) was compared to understand the role of proteinase K. We found that 10 cfu mL^−1^ of CI cells could grow to 1.9 × 10^5^ cfu mL^−1^ in FM and 5.2 × 10^5^ cfu mL^−1^ in FM + PK (Figure 2a). The addition of proteinase K could support only 2.7-fold more growth in culturable cells, but this difference in growth was not statistically significant. So, proteinase K itself did not act as a nutrient source. Similarly, 0.04 cfu mL^−1^ of VBNC cells showed no recovery in FM and could attain a recovered cell count up to 1.4 ± 0.5 × 10^7^ cfu mL^−1^ in FM + PK (Figure 2a). Therefore, spent ASW was indispensable for resuscitation to occur in VBNC cells of *V. cholerae*.

Next, the growth of culturable and VBNC cells was compared in spent ASW media (SM) and SM+PK to understand the role of proteinase K. The culturable cells could grow to 2.45 × 10^6^ cfu mL^−1^ in SM and 4.3 × 10^7^ cfu mL^−1^ in SM + PK (Figure 2b). The growth of culturable cells was 13-fold higher in SM than FM and 17.5-fold higher in SM+PK than SM. This indicates that spent ASW media could provide nutrients for growth, and the addition of proteinase K further enhanced its growth-promoting ability. Likewise, we observed that VBNC cells could grow up to 4.9 × 10^6^ cfu mL^−1^ in SM and 1.58 × 10^8^ cfu mL^−1^ in SM+PK (Figure 2b). The growth of VBNC cells was 32-fold higher in SM+PK than SM. These data further showed that the growth-promoting effect of proteinase K was higher for stressed VBNC cells compared to culturable cells.

### 3.3. Time-Dependent Recovery of VBNC Cells and Growth of Culturable Cells

**Figure 3:**
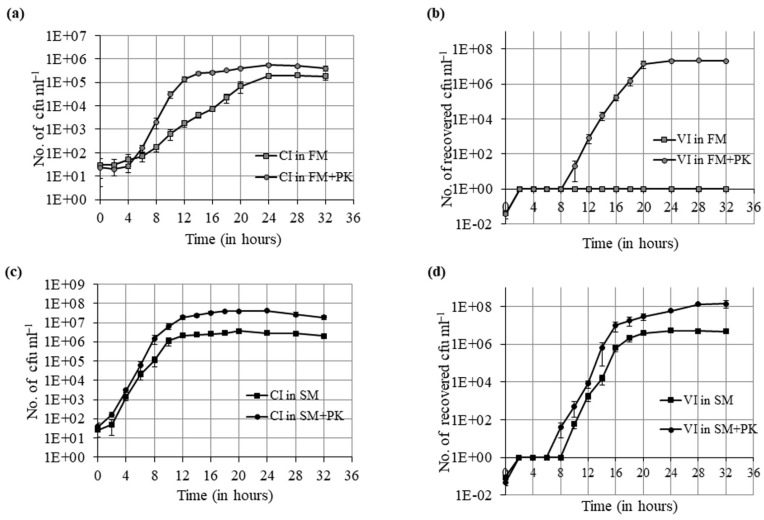
Time-dependent growth curve of culturable cells and resuscitation curve of VBNC cells in ASW (FM), ASW with proteinase K ( FM + PK), spent ASW (SM) and spent ASW with proteinase K (SM + PK). (**a**) Growth curve of culturable cells (CI cells) in FM (■) and FM + PK (●) for 32 h at 37 °C. The viable cell count was determined by the plate count method at an interval of 2 h until the end of incubation time. (**b**) Resuscitation curve of VBNC cells (VI cells) in FM (■) and FM + PK (●) for 32 h at 37 °C. (**c**) Growth curve of CI cells in SM (■) and SM+PK (●) for 32 h at 37 °C. (**d**) Resuscitation curve of VI cells in SM (■) and SM+PK (●) for 32 h at 37 °C. Each data point represents the mean ± SE of three independent experiments.

We examined the time-dependent growth of culturable cells in ASW media and proteinase K-supplemented ASW media. The culturable cells could grow from 10 cfu mL^−1^ to 10^5^ cfu mL^−1^ in FM as well as in FM + PK, but the time required to attain that cell number was different. The culturable cells took 12 h to reach 10^5^ cfu mL^−1^ in FM + PK with a generation time (gt) of 38.5 min, whereas they needed more than 20 h in FM with a gt of 86.6 min (Figure 3a and Figure S1a,b). Similarly, the time-dependent growth of VBNC cells in ASW media and proteinase K-supplemented ASW media was examined. As mentioned previously, VBNC cells were not able to grow at all in ASW media. However, 10 cfumL^−1^ of recovered cells was observed in FM + PK after a lag period of approximately 8 h. Then, it could grow to reach 10^5^ cfu mL^−1^ in 6 h, whereas culturable cells took twice this time duration to reach an equivalent number of cells in FM + PK. These recovered cells further grow to 10^7^ cfu mL^−1^ in another 4 h. So, VBNC cells showed maximum growth in a total time duration of 10 h with a gt of 31.5 min (Figure 3b and Figure S1c). The obtained results showed that the growth rate of culturable cells was increased with proteinase K but the impact of proteinase K was higher on the growth rate of recovered cells from the VBNC state.

Next, the effect on the growth rate of culturable and VBNC cells was determined by growth curve analysis in spent ASW media and proteinase K-supplemented spent ASW media. The culturable cells took 10 h to reach 10^6^ cfu mL^−1^ in SM with a gt of 34.6 min and 10^7^ cfu mL^−1^ in SM+PK with a gt of 31.5 min (Figure 3c and Figure S2a,b). So, the effect of proteinase K on the growth rate of culturable cells in spent ASW media was not as prominent as in ASW media. VBNC cells showed an initial lag of 8 h in spent ASW media before it could reach 10 cfu mL^−1^, and in the next 10 h, it reached 10^6^ cfu mL^−1^ with a gt of 31.5 min (Figure 3d and Figure S2c). This implies that a small population of VBNC cells undergo recovery during the initial non-growth phase and then grow like culturable cells. Moreover, we obtained a detectable number of recovered cells in proteinase K-supplemented spent ASW after 6 h of lag period, and these recovered cells reached 10^7^ cfu mL^−1^ with a gt of 26.6 min (Figure 3d and Figure S2d). So, the addition of proteinase K could shorten the lag period, resulting in a faster transition from VBNC to recovery states.

### 3.4. Effect of Serine Protease on Recovery of V. cholerae from VBNC State Is a General Trait

**Figure 4:**
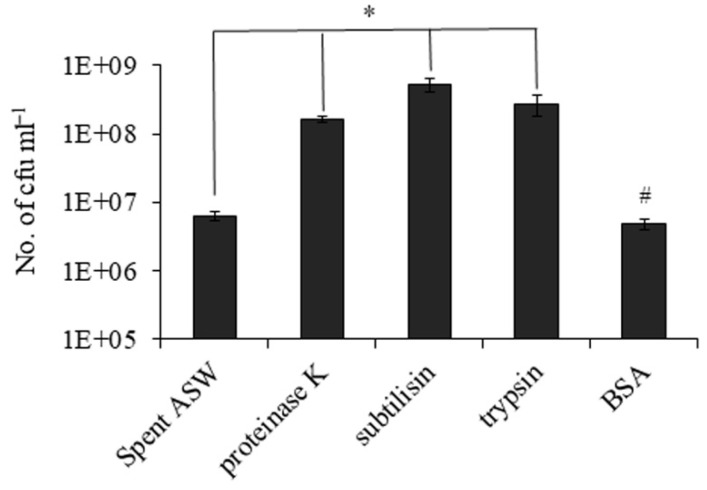
Effect of protease on recovery of *V. cholerae* from the VBNC state is a general trait. Recovery was induced in VBNC cells at 37 °C in spent ASW media (SM) and heat-treated spent ASW media (∆SM) without or with proteinase K, subtilisin, trypsin and BSA. The recovered cell count was determined after 32 h. Asterisk (*) represents statistically significant difference in growth of VBNC cells with and without proteases in spent ASW media (*p* < 0.05). # represents non-significant difference in growth of VBNC cells with and without proteases in heat-treated spent ASW media.

Next, the effect of other serine proteases such as trypsin and subtilisin was also tested on the recovery from the VBNC state. We inoculated 0.02 to 0.08 cfu mL^−1^ of VBNC cells in spent ASW media supplemented with proteinase K, subtilisin, trypsin and BSA followed by incubation at 37 °C for 32 h. The numbers of recovered cells were 6.2 × 10^6^, 1.67 × 10^8^, 5.3 × 10^8^, 2.8 × 10^8^ and 4.8 × 10^6^ cfu mL^−1^ for VBNC only, with proteinase K, with subtilisin, with trypsin and with BSA, respectively (Figure 4). So, the effect of proteinase K on recovery was not an exclusive property; other serine proteases also had similar effects.

### 3.5. Growth-Promoting Components of Spent ASW Media

**Figure 5:**
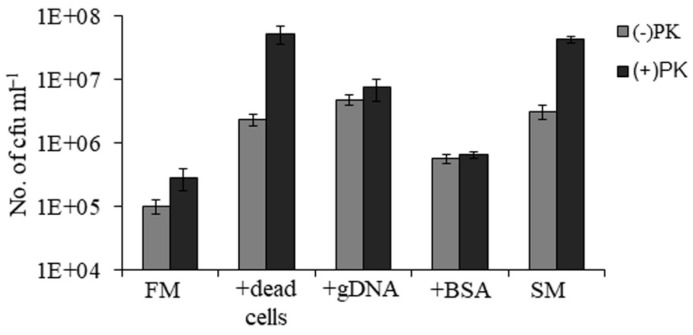
Growth-promoting components of spent ASW media. The growth of culturable cells was checked in ASW media (FM), ASW media with 10^7^ cells of dead bacteria (+dead cells), ASW media with 10 µg mL^−1^ genomic DNA (+gDNA), ASW media with 100 of µg mL^−1^ BSA (+BSA) and spent ASW media (SM). The cell count was determined after 32 h of incubation and represented as (−) PK. The samples in the presence of proteinase K are represented as (+) PK.

The components of spent ASW media which might support the growth of VBNC cells were dead bacteria, genomic DNA and proteins. So, we used 10^7^ cells of dead bacteria, 10 µg mL^−1^of genomic DNA and 100 µg of mL^−1^ BSA as nutrient sources and added them to ASW media to check the effect of these components on the growth of culturable cells. The numbers of viable cells obtained after 32 h in FM, FM with dead cells, FM with genomic DNA, FM with BSA and spent ASW media were 1 × 10^5^, 2.4 × 10^6^, 4.8 × 10^6^, 5.7 × 10^5^ and 3.1 × 10^6^ cfu mL^−1^, respectively (Figure 5). This result showed that the addition of dead cells or genomic DNA in nutrient-deficient ASW media could support the growth of culturable cells. However, ASW media supplemented with either dead cells or genomic DNA was incapable of supporting the transformation of VBNC cells into recovered cells. It was only the addition of proteinase K in ASW media that led to recovery in VBNC cells as mentioned earlier. Therefore, protease might play an essential role during the process of resuscitation.

Similarly, this experiment was also performed in the presence of proteinase K. The numbers of viable cells obtained in FM, FM with dead cells, FM with genomic DNA, FM with BSA and spent ASW media were 6 × 10^5^, 5.3 × 10^7^, 7.5 × 10^6^, 6.5 × 10^5^ and 4.3 × 10^7^cfu mL^−1^, respectively (Figure 5). The ASW media supplemented with dead cells and proteinase K could support a growth rate equivalent to spent ASW media supplemented with proteinase K. This implies that dead cells were present in spent ASW media on which proteinase K might act to support the high growth of recovered cells.

### 3.6. The Recovery State Shows an Increase in Protease Expression

**Figure 6:**
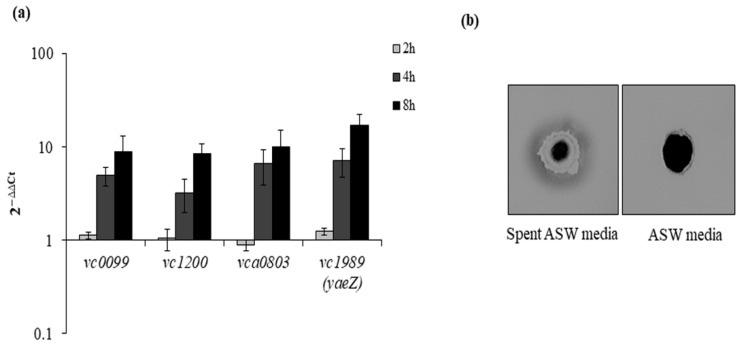
Protease expression at mRNA and protein level during recovery. (**a**) We checked the mRNA expression of serine proteases such as *vc0099*, *vc1200*, *vca0803* and *vc1989* (*yaeZ*) in a time-dependent manner (4 h, 8 h, 16 h) using *dnaK* as reference gene. (**b**) Skim milk plate assay for the detection of protease secretion during recovery from VBNC state. Protease activity on 1% skim milk plate.

The effect of proteinase K on the growth of *V. cholerae* during recovery prompted us to check the mRNA expression of serine proteases such as *vc0099*, *vc1200*, *vca0803* and *vc1989* (*yaeZ*) in a time-dependent manner. We found that each of the serine proteases showed a gradual increase in mRNA expression (Figure 6a). This suggests that protease might play a crucial role in the resuscitation of bacteria from a dormant state followed by growth. In another experiment, the protease expression of VBNC cells was checked by skim milk plate assay. We prepared two types of 1% skim milk plate: one plate was prepared using ASW media, and the second one was prepared using spent ASW media. We found that the VBNC sample showed growth and distinct clear zones only around the well in the skim milk plate constituted of spent ASW media (Figure 6b).

## 4. Discussion

The dormant VBNC state can be transitioned to an active culturable state by several methods such as high temperature, the addition of nutrients, catalase and direct interaction with eukaryotic cells [3,9,13,14]. This study showed that spent medium filtered from VBNC microcosm was indispensable for the temperature-dependent recovery of VBNC cells. It was shown in *Staphylococcus aureus* that spent media could induce recovery in dormant cells, and the resuscitation effect of spent culture was greatly reduced by boiling or the addition of 50 µg mL^−1^ trypsin [23]. Similarly, it was observed that the heat treatment could destroy the resuscitation-promoting trait of spent media. However, the addition of proteases such as proteinase K, trypsin or subtilisin in spent media showed different effects on *V. cholerae* in the present study. Instead of inhibiting the recovery from the VBNC state, the addition of these serine proteases in spent ASW media led to more efficient recovery compared to the recovery dependent only upon higher temperature. So, whether it was trypsin-like or subtilisin-like protease, the overall effect on recovery was similar. This might be because the substrate on which these serine proteases acted was the same and thus similar results were produced. Therefore, it seems to be a general trait of serine protease to enhance the resuscitation-promoting effect of spent ASW during recovery. Further investigation on other Gram-negative bacteria such as *E. coli* is required to get a more detailed insight into the role of serine protease during recovery from the VBNC state.

Therefore, in order to understand the effect of proteinase K only, ASW media supplemented with proteinase K was used instead of spent media for the induction of recovery. Interestingly, in ASW media, which otherwise could not support the recovery of VBNC cells, the presence of proteinase K made that possible. So, even if we assume that this growth was due to the inclusion of few undetected culturable cells, as also suggested by Ravel et al. [7], it is quite intriguing that VBNC cells could grow up to 10^7^ cfu mL^−1^ in nutrient-deficient ASW media by the mere addition of proteinase K. The mode of action of proteinase K was analyzed by comparing the growth of culturable and VBNC cells either in the absence or presence of proteinase K in a time-dependent growth manner. In the presence of proteinase K, the culturable cells showed a significant increase in the growth rate by reducing the generation time, and the growth rate of VBNC cells was faster with proteinase K than culturable cells. It was reported that Rpfs of Mycobacteria could stimulate cell division, either by the remodeling of the cell wall, or cell lysis products could initiate an anti-dormancy signaling cascade [26]. It might be speculated that proteinase K acts in a similar way.

In addition, the comparison of the time-dependent recovery of VBNC cells between spent ASW and proteinase K-supplemented spent ASW media showed that proteinase K could accelerate the transition from VBNC to recovered cells, and these few recovered cells then grew like culturable cells but with a higher growth rate. We speculate that this increased growth rate of recovered cells was probably due to the availability of an ample amount of pre-digested (by proteinase K) cellular components present in spent ASW media. The shortening of the lag phase in the presence of Rpfs was also reported in *S. aureus* [23]. Furthermore, the expression level of serine proteases was examined during the course of recovery. Increased mRNA expression of serine proteases such as *vc0099, vc1200, vca0803* and *vc1989* (*yaeZ*) was found. A recent study showed that the proteolytic activity of YeaZ promotes resuscitation in VBNC cells of *V. harveyi,* which might be due to the stimulation of the cell division process in the VBNC cells [22]. This study showed evidence that proteinase K might play a similar role during the resuscitation of VBNC cells of *V. cholerae*. It had also been reported that the addition of YaeZ could result in a higher number of recovered cells and also could induce resuscitation beyond the recovery end point [27]. The nucleotide sequence of *V. cholerae* YaeZ showed 70% identity with that of *V. harveyi* and *V. parahaemolyticus*. The present study found a similar observation with proteinase K, where the addition of proteinase K at the time of temperature transition from 4 °C to 37 °C led to an extension in the total recovery period from a period of 2 weeks to 5 weeks and a higher number of total recovered cells.

Moreover, genomic DNA and dead bacterial cells present in spent ASW media were identified as good nutrient sources that could be utilized by *V. cholerae* cells for growth [28,29]. However, neither the addition of dead cells nor genomic DNA in ASW media could induce resuscitation in VBNC cells. This indicates that their role might be limited to supporting the growth of few recovered cells rather than the induction of resuscitation. Among them, dead cells present in spent ASW seem to be the substrate for proteinase K to support the high growth of recovered cells. A recent study showed that the staphylococcal proteases aureolysin and staphopain B cleave collagen into peptide fragments that support *S. aureus* growth under nutrient-limited conditions inside a host [30]. So, it can be speculated that proteinase K might act on the membrane protein of dead bacteria to release peptides, which ultimately support the growth of few recovered cells.

The vast majority of environmental microbes are not capable of growth on laboratory culture media, either due to a lack of a customized culture technique or because of dormancy. So, it is important to develop novel cultivation techniques. Recently, external stimulants have been successfully used to revive bacteria from environmental sources. Su et al. reported that the isolation of biphenyl-degrading bacteria from PCB-contaminated soils was enhanced with the addition of Rpf-containing culture supernatant from *M. luteus* [31]. In another study, an enrichment media was developed that consisted of 10 mmol L^−1^ sodium pyruvate in low-nutrient medium, and it was used successfully for the isolation of previously uncultured bacteria from the coastal sediment of China [32]. Similarly, an enrichment media might be formulated for *V. cholerae* using minimal media supplemented with proteinase K. The environmental water samples should be first enriched in this medium, followed by plating on commercially available selective media for Vibrios.

Altogether, this study highlighted the role of protease during recovery from the VBNC state, whether produced by the bacteria or supplemented. The present data project that serine protease could be a possible choice for a resuscitation-promoting factor in *V. cholerae*. Thus, we propose that after a temperature upshift, transition from a VBNC to a recovery or culturable state might occur during the initial lag phase of 6–8 h. The presence of proteinase K could shorten the length of the lag phase and expedite the transition phase by activating the cell division process. Then, the recovered cells could grow rapidly in nutrient-rich spent ASW media to achieve a higher cell count. Proteinases are found in all domains of life, and one-third of them are serine proteases. In humans, serine proteases are constitutively produced by the pancreas, epithelial cells, immune and mesenchymal cells, whereas serine proteases were mainly studied as virulence factors among bacterial pathogens such as *V. cholerae*. It might be possible that VBNC cells are reactivated and multiplied through the assistance of host and bacterial proteases inside the intestinal environment of humans. Thus, detailed insights into the resuscitation of VBNC cells is essential for the better management of this highly infectious human pathogen which often remains undetected in environmental samples.

## Figures and Tables

**Table 1:** Resuscitation-promoting ability of VBNC cells in different media.

Sample	Recovered cfu mL^−1^
Undiluted VBNC	6 × 10^6^
10-fold diluted VBNC in spent ASW media	4.9 × 10^6^
10-fold diluted VBNC in heat treated spent ASW media	No recovery
10-fold diluted VBNC in spent ASW supplemented with Proteinase K	1.58 × 10^8^

## Supplementary Materials

The following are available online at https://www.mdpi.com/article/10.3390/microorganisms9122618/s1, Figure S1. (a), (b) The calculation of generation time (gt) of culturable (CI) cells in ASW media (FM) and proteinase K-supplemented ASW media ( FM + PK). The data points that represent the log phase from the growth curve were used to determine the generation time for CI cells in FM and FM + PK media. All the experiments were performed thrice, and each data point represents the mean ± SE. (c) The calculation of generation time (gt) of VBNC (VI) cells in FM + PK. The data points which represent the log phase from the growth curve were used to determine the generation time for VI cells in FM + PK media. All the experiments were performed thrice, and each data point represents the mean ± SE. Figure S2. (a), (b) The calculation of generation time (gt) of culturable (CI) cells in spent ASW media (SM) and proteinase K-supplemented spent ASW media (SM + PK). The data points which represent the log phase from the growth curve were used to determine the generation time for CI cells in SM and SM + PK media. All the experiments were performed thrice, and each data point represents the mean ± SE. (c), (d) The calculation of generation time (gt) of VBNC (VI) cells in SM and SM + PK. The data points which represent the log phase from the growth curve were used to determine the generation time for VI cells in SM and SM + PK media. All the experiments were performed thrice, and each data point represents the mean ± SE. Table S1. List of primers used in this study.

## Abbreviations

VBNC	viable but non-culturable
ASW	artificial sea water
FM	ASW media
SM	spent ASW media
PK	proteinase K
FM + PK	proteinase K-supplemented ASW media
