# Supplementary material for: The Impact of Protease during Recovery from Viable but Non-Culturable (VBNC) State in Vibrio cholerae
Source: Microorganisms. 2021 Dec 18;9(12):2618. doi: 10.3390/microorganisms9122618 (PMC8707003; doi:10.3390/microorganisms9122618)
Supplement: Supplementary file 1 [file microorganisms-09-02618-s001.zip › microorganisms-1464862-supplementary.pdf]

microorganisms

Supplementary Material

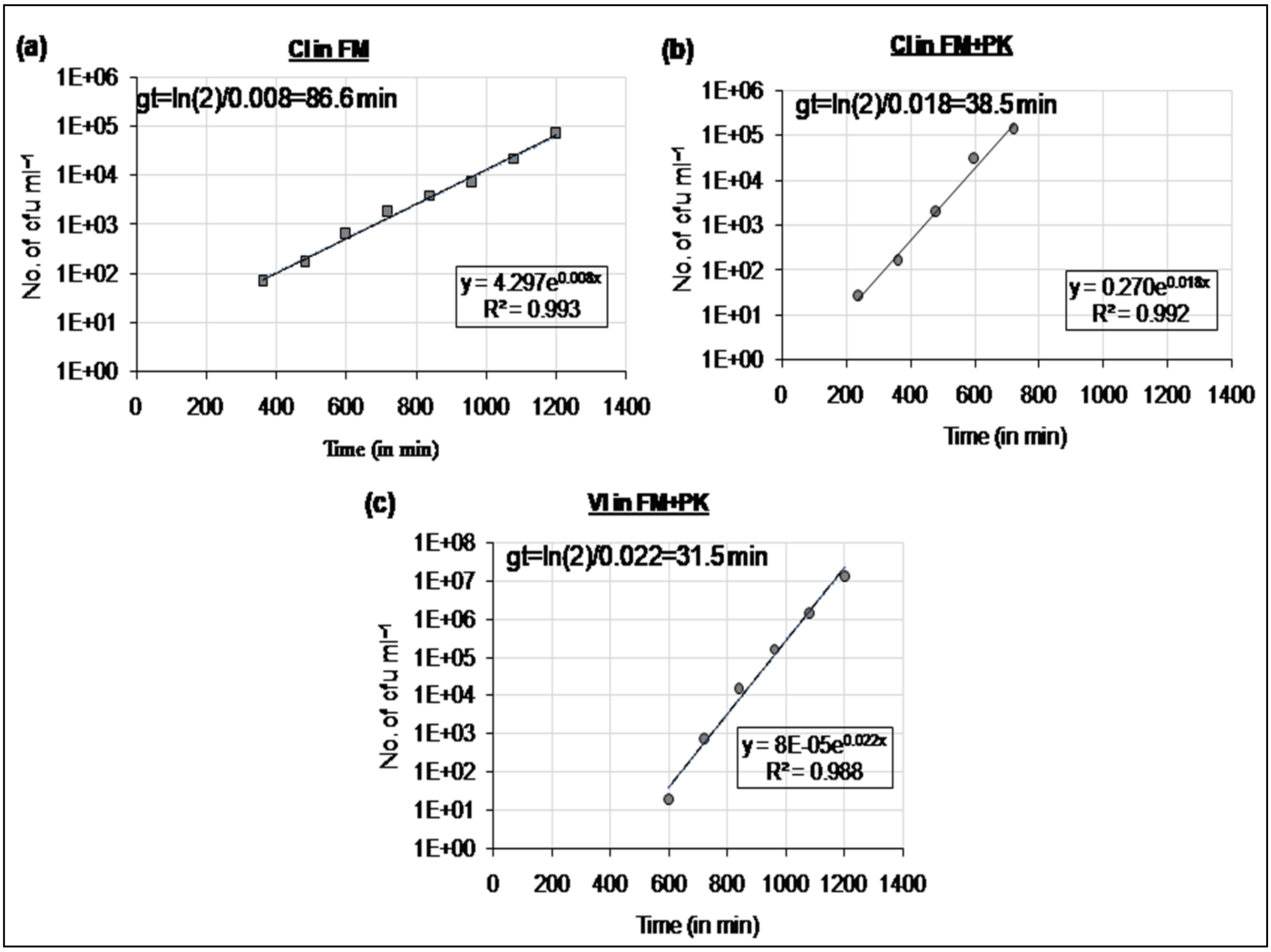

Figure S1. (a), (b) The calculation of generation time (gt) of culturable (CI) cells in ASW media (FM) and proteinase K supplemented ASW media (FM+PK). The data points which represents log phase from growth curve were used to determine the generation time for CI cells in FM and FM+PK media. All the experiments were performed thrice and each data point represents the mean ± SE. (c) The calculation of generation time (gt) of VBNC (VI) cells in FM+PK. The data points which represents log phase from growth curve were used to determine the generation time for VI cells in FM+PK media. All the experiments were performed thrice, and each data point represents the mean ± SE.

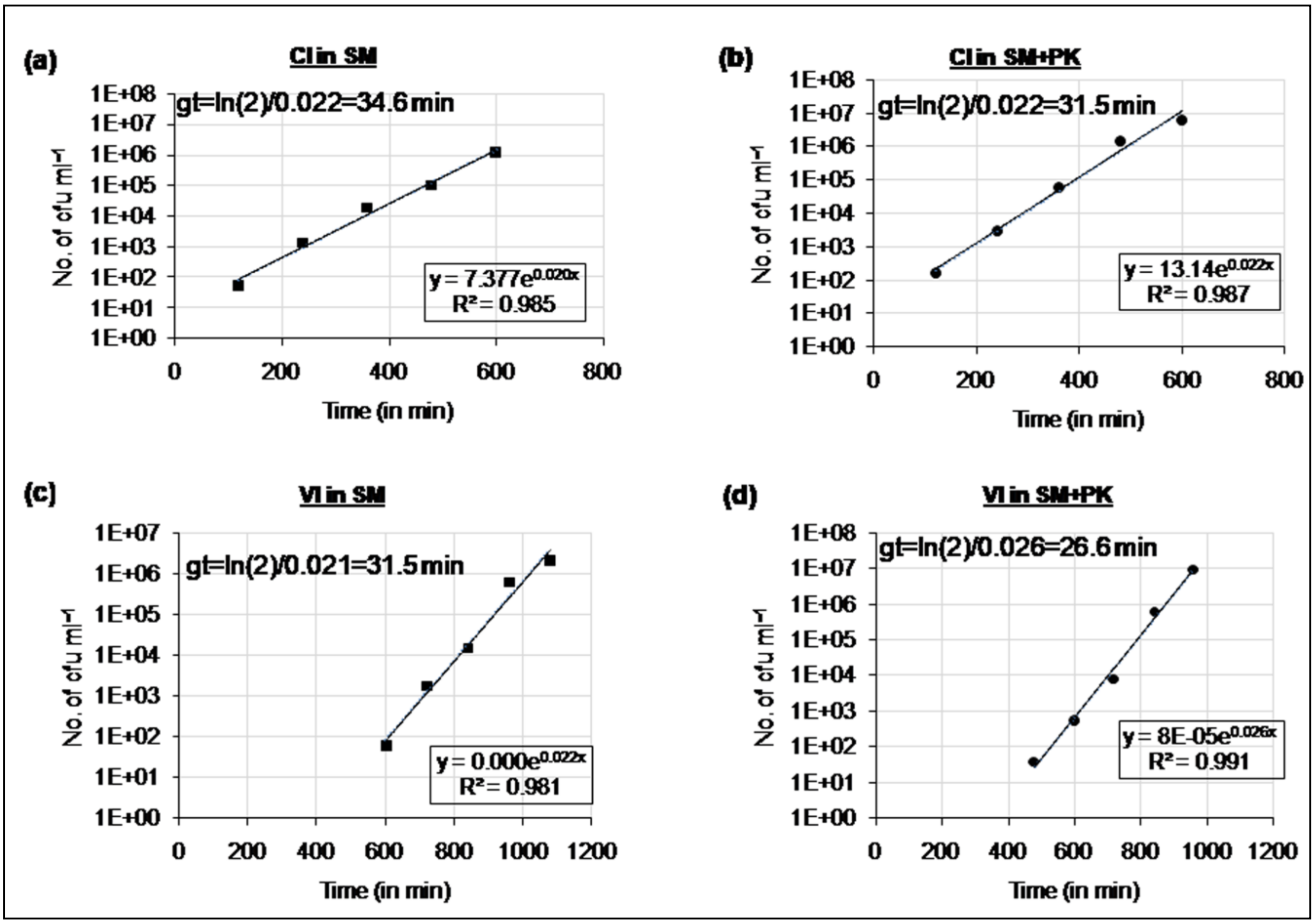

Figure S2. (a), (b) The calculation of generation time (gt) of culturable (CI) cells in spent ASW media (SM) and proteinase K supplemented spent ASW media (SM+PK). The data points which represents log phase from growth curve were used to determine the generation time for CI cells in SM and SM+PK media. All the experiments were performed thrice and each data point represents the mean ± SE. (c), (d) The calculation of generation time (gt) of VBNC (VI) cells in SM and SM+PK. The data points which represents log phase from growth curve were used to determine the generation time for VI cells in SM and SM+PK media. All the experiments were performed thrice, and each data point represents the mean ± SE.

Table S1. List of primers used in this study.

Gene locus	Primer	Sequence (5′→3′)
vc0099	vc0099_Fo	CCATAAACGGCTGCACATAAC
	vc0099_Rv	CTATGCTTTGGCTGGGTATCT
vc1200	vc1200_Fo	TCGAGTTAGCACAAACCAGTAG
	vc1200_Rv	CCCATCCAGCCACTGTTAAA
vca0803	vca0803_Fo	ACTGCCGAAGAATCGACATAG
	vca0803_Rv	GTGGTTCCTCAGTTGGAAGAT
vc1989	vc1989_Fo	CTGCTCATCTTGGGTTAAATCATC
	vc1989_Rv	GCGCATGGAAGAAGTGTATTG
dnaK	dnaK_Fo	TACTGCAAGGTGAGCGTAAG
	dnaK_Rv	GTCTGCATCCAAGTCGAAGATA
groEL	groEL_Fo	CGCTTCTCCTTCCACATCTT
	groEL_Rv	CATCCTGCTGGTGGATAAGAA
